# Goodpasture's syndrome with positive C-ANCA and normal renal function: A case report

**DOI:** 10.1186/1752-1947-2-223

**Published:** 2008-06-30

**Authors:** Arunachalam Ramaswami, Thiraviyam Kandaswamy, Tholappan Rajendran, Hla Aung, Chakko K Jacob, Haji Shaukat Zinna, Pemasiri Upali Telesinge

**Affiliations:** 1 Department of Nephrology, RIPAS Hospital, Brunei Darussalam; 2 Department of Pathology, RIPAS Hospital, Brunei Darussalam

## Abstract

**Introduction:**

Goodpasture's syndrome consists of a triad of pulmonary hemorrhage, rapidly progressive glomerulonephritis and anti-glomerular basement membrane (anti-GBM) antibodies, either in circulation or fixed to the kidney. The absence of renal manifestations is uncommon. We present a case of biopsy proven anti-GBM antibody disease with normal renal function, mild urinary abnormalities and positive C-antineutrophil cytoplasmic antibody (C-ANCA) serology.

**Case presentation:**

A 44-year-old female was treated for repeated episodes of hemoptysis and one episode of respiratory failure requiring ventilatory support. She had minor urinary abnormalities in the form of microscopic hematuria and non-nephrotic proteinuria. She also had positive C-ANCA. Her lung biopsy showed evidence of intra-alveolar hemorrhage with linear IgG deposits in the basement membrane of the alveolar capillaries. Owing to these lung biopsy findings, a kidney biopsy was carried out, which showed minimal thickening of the glomerular basement membrane and linear IgG and C3 deposits along the capillary walls. Her renal function remained persistently normal.

**Conclusion:**

Goodpasture's syndrome is a rare disease. Even though the classical presentation is that of rapidly progressive glomerulonephritis pulmonary hemorrhage and anti-GBM antibodies in the circulation and kidneys, in rare cases it can present with repeated pulmonary hemorrhage and minor urinary abnormalities. In all cases of repeated pulmonary hemorrhage, the possibility of Goodpasture's syndrome should be considered and investigated further.

## Introduction

The eponym Goodpasture's syndrome has been offered to patients with pulmonary hemorrhage (hemoptysis) and glomerulonephritis (hematuria) along with circulating anti-glomerular basement membrane (anti-GBM) antibodies [1]. The auto-antibodies are directed against the Goodpasture antigen, which is part of the non-collagenous domain of the alpha 3(IV) collagen chain. It is an uncommon condition and is often fatal owing to uncontrollable pulmonary hemorrhage or rapidly progressive renal failure. There have been occasional reports of individuals with Goodpasture's syndrome and normal renal function, either as individual cases or within a series [2]. Here we report a patient with repeated hospitalization for pulmonary hemorrhage with microscopic hematuria and normal renal function. Renal biopsy showed linear IgG deposits on immunoflorescence. The patient also tested positive for C-antineutrophil cytoplasmic antibody (C-ANCA).

## Case presentation

A 44-year-old female was hospitalized on 25th January 2002 for shortness of breath and hemoptysis. Her past medical history consisted of pulmonary tuberculosis treated in 1983 and thyroid surgery for thyrotoxicosis in 1991. She had an episode of hemoptysis in 2001. She was labeled as recurrent acute respiratory distress syndrome (ARDS) in 2002.

On examination at the time of admission her respiratory rate was 30/min, pulse rate 120/min and blood pressure (BP) was 170/80. Chest X-ray (CXR) showed diffuse parenchymal lesions with fibrosis of both lungs. Her oxygen saturation dropped to 72% and she required mechanical ventilation. Sputum culture grew *Klebsiella pneumoniae *and hence she was treated with Amoxicillin clavalunic acid and Ceftazidime. Following this treatment her clinical condition improved and she was discharged.

She again presented in February 2003 with another episode of hemoptysis. This time a chest computed tomography (CT) scan showed diffuse interstitial fibrosis mostly in both apices. There was a ground glass appearance.

Her fourth episode of hemoptysis occurred when she presented to the hospital in July 2006 with shortness of breath. During this admission her pulmonary function tests showed combined obstructive and restrictive changes. Bronco-alveolar lavage showed hemorrhagic effluent with a large number of hemosiderin containing histiocytes, few lymphocytes and polymorphonuclear leucocytes.

Her investigations revealed a urine protein level of 2+ and a red blood cell count of 10–20/HPF, while blood examination showed a white blood cell count of 15.6 × 10^3^/μl, hemoglobin at 12.4 g/dl and a platelet count of 301 × 10^9^/l. Blood urea was at 2.4 mmol/l and S-creatinine was at 48 μmol/l. Rheumatoid factor test was positive at 32 IU, as was an autoantibodies test at 1/160. Anti-ds DNA negative C-ANCA was weakly positive. Her shortness of breath increased. A chest CT scan showed extensive ground glass appearance. Anti-GBM antibodies were negative.

### Lung biopsy findings [Fig 1]

**Figure 1:**
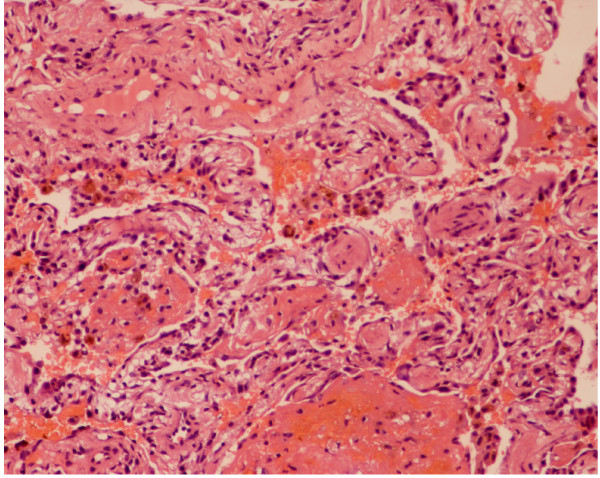
**Photomicrograph of a lung biopsy demonstrating hyaline masses lined by type II pneumocytes and blood in the alveolar spaces**. Hematoxylin and eosin stain.

An open wedge biopsy from the lower lobe of the right lung was carried out. This showed marked intra-alveolar hemorrhage with the presence of a large number of siderophages. A prominent proliferation of type 2 pneumocytes was also seen. Alveolar septae were inflamed, thickened and fibrotic with the formation of collagenous nodules. Immunoperoxidase staining showed linear deposition of IgG in the basement membrane of several alveolar capillaries with intra-alveolar hemorrhage and septal fibrosis.

### Kidney biopsy [Fig 2 and Fig 3]

**Figure 2:**
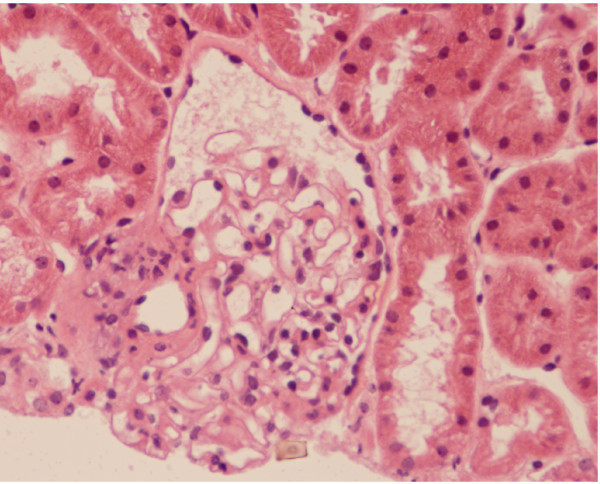
**Photomicrograph of a kidney biopsy demonstrating minimal thickening of the glomerular basement membrane and increased cellularity**. Hematoxylin and eosin stain.

**Figure 3:**
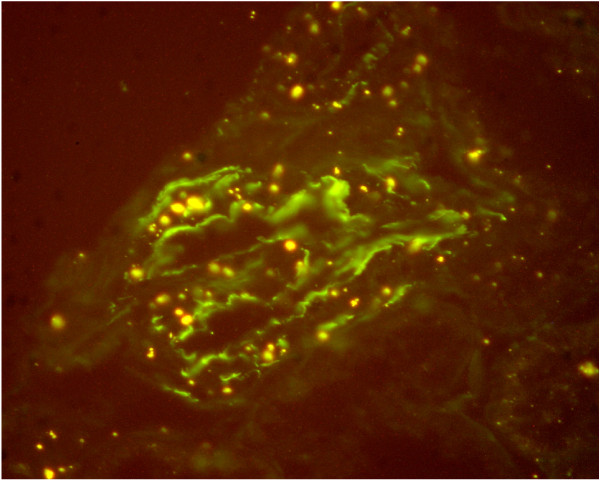
**Photomicrograph of a kidney biopsy**. Immunoflorescense staining showing IgG and C3 along capillary walls.

In view of the recurrent pulmonary hemorrhage with proteinuria and microscopic hematuria despite normal renal function, the patient was offered a renal biopsy to which she consented in November 2006. Renal biopsy was assessed by light and immunoflorescence microscopy. The light microscopy showed eight glomeruli with minimal thickening of the basement membrane with increased cellularity. Immunoflorescence showed linear staining of IgG and C3 along the capillary walls. The pathology report was consistent with anti-GBM antibody disease.

The patient commenced treatment with oral Prednisolone 30 mg/day and oral Cyclophosphamide 100 mg/day. She has been on outpatient follow-up since August 2006 and is doing well on this treatment. Her latest investigations were: urine protein negative, red blood cell count 10/μl, white blood cell count 6/μl, hemoglobin 15.1 g/dl, platelet count 251/l, blood urea 2.5 mmol/l, Na 137.7 mmol/l, K 3.83 mmol/l, creatinine 68 μmol/l total protein 71 g/l, alb 34 g/l, and uric acid 312. She has had no relapse of hemoptysis and is free from respiratory symptoms.

## Discussion

Goodpasture's syndrome is rare and its incidence has been estimated to be 0.5% new cases/million/year in the UK. It has a poor prognosis in spite of treatment with mortality of 11% and high morbidity. Some 60% of patients become dependent on dialysis. Most cases of Goodpasture's syndrome have both pulmonary and renal manifestation and often there is extensive and progressive glomerular disease. Patients have marked renal insufficiency and severe crescentic glomerulonephritis with crescents involving a mean of 77% glomeruli in one report [2].

In the case presented here, in common with other cases of Goodpasture's syndrome, there was deterioration of pulmonary function and hemoptysis in the presence of chest infection. A rare and unusual finding in the present case was little evidence of clinical renal disease despite the presence of florid pulmonary disease. Kidney biopsy, however, showed linear deposits of IgG in most of the glomeruli.

Another interesting finding is the C-ANCA positivity. In one study, positive ANCA was seen in 21.3% of 160 patients with Goodpasture's syndrome. In the setting of anti-GBM disease, ANCA seropositivity has important clinical and prognostic implications. These patients may have extra-renal and extra-pulmonary manifestations. They are more likely to develop recurrent renal or pulmonary disease and have a more favorable prognosis [3].

This patient's history highlights the important point that physicians need to consider the diagnosis of Goodpasture's syndrome in patients with pulmonary hemorrhage even when the renal function is normal and the urine shows only minor abnormalities such as microscopic hematuria. Whether these cases represent a distinct form of Goodpasture's syndrome or simply the same disease at an earlier stage is a moot point. Under normal circumstances the alveolar capillaries are continuous unlike glomerular capillaries. Large charged molecules such as immunoglobulin are excluded. It is only after damage to the lungs (for example, as a result of infection or cigarette smoking) that the alveolar capillaries become more porous and the alveolar Goodpasture antigen becomes exposed. It is perhaps this unusual exposure in the presence of infection or damage which triggers the autoimmune process in genetically susceptible individuals.

There may be variations in the pathological mechanism by which variants of anti-GBM disease may arise. Some studies have revealed that the antibodies to the NCl epitopes are found universally in Goodpasture's syndrome Additional limited reactivity to other epitopes, for example, alpha (4) NCl or alpha 4(IV) NCl is found in a minority of individuals (15%). Moreover, non-alpha 3(IV) epitope reactivity was found in patients with anti-GBM disease and glomerulonephritis alone [4].

In one study, circulating anti-GBM antibodies were detected less often and at lower levels in individuals with normal renal function than in those with impaired renal function. About 15% of patients with anti-GBM disease have no circulating antibodies at all. This is explained by the antibody being removed from the plasma by binding to the kidneys, by the GBM used in the assay lacking the antigen found in native GBM or by circulating anti-GBM antibodies being of lower affinity than those that are tissue bound. Lower levels or the absence of circulating anti-GBM antibodies may correlate with better preserved renal function [5].

## Conclusion

Anti-GBM antibody disease has to be considered in patients presenting with pulmonary hemorrhage or anemia despite normal renal function and minor urinary abnormalities. Despite minor urinary abnormalities and normal renal function, kidney biopsy may show extensive linear deposits in the glomeruli. Lung biopsy may be required for the diagnosis of Goodpasture's syndrome in those with minor renal abnormalities [6]. Goodpasture's syndrome with normal renal function may be an early form of the disease. It can be treated with immunosuppression.

## Competing interests

The authors declare that they have no competing interests.

## Consent

Written informed consent was obtained from the patient for publication of this case report and accompanying images. A copy of the written consent is available for review by the Editor-in-Chief of this journal.

## Authors' contributions

AR, TK, TR, HA, CKJ, HSZ contributed to the preparation of manuscript and PUT processed the lung biopsy and kidney biopsy specimens and prepared the biopsy reports.
